# Safety and efficacy of self‐administered romiplostim in patients with immune thrombocytopenia: Results of an integrated database of five clinical trials

**DOI:** 10.1002/ajh.25776

**Published:** 2020-03-21

**Authors:** David J. Kuter, Donald M. Arnold, Francesco Rodeghiero, Ann Janssens, Dominik Selleslag, Robert Bird, Adrian Newland, Jiri Mayer, Kejia Wang, Robert Olie

**Affiliations:** ^1^ Hematology Division Massachusetts General Hospital Boston Massachusetts USA; ^2^ Canadian Blood Services and Department of Medicine, McMaster Centre for Transfusion Research Michael G DeGroote School of Medicine, McMaster University Hamilton Ontario Canada; ^3^ Haematology Project Foundation, Affiliated to the Department of Haematology S. Bortolo Hospital Vicenza Italy; ^4^ Department of Hematology University Hospitals Leuven Campus Gasthuisberg, Leuven Belgium; ^5^ Department of Hematology AZ Sint Jan Brugge Bruges Belgium; ^6^ Division of Cancer Services Princess Alexandra Hospital Brisbane Australia; ^7^ The Pathology Clinical Academic Group The Royal London Hospital London UK; ^8^ Department of Internal Medicine, Haematology and Oncology Masaryk University and University Hospital Brno Brno Czech Republic; ^9^ Amgen Inc Thousand Oaks California USA; ^10^ Amgen (Europe) GmbH Rotkreuz Switzerland

## Abstract

Romiplostim self‐administration by patients or caregivers may offer time/cost savings to healthcare professionals (HCPs) and convenience for patients who avoid weekly clinic visits. We performed an integrated analysis of five clinical trials to evaluate the efficacy and safety of romiplostim self‐administration. Data were analyzed from adults with immune thrombocytopenia (ITP) who received weekly romiplostim via self‐administration or from an HCP. Patients who achieved a stable romiplostim dose for ≥3 weeks (HCP group ≥5 weeks to provide an appropriate index date to enable comparisons with the self‐administration group) with platelet counts ≥50 × 10^9^/L were eligible. In the self‐administration (n = 621) vs HCP (n = 133) groups, respectively, median age was 53 vs 58 years, median time since primary ITP diagnosis was 3.7 vs 2.5 years, and median baseline platelet count at ITP diagnosis was 19.0 vs 20.0 × 10^9^/L. In the self‐administration and HCP‐dosed groups, median romiplostim treatment duration was 89 vs 52 weeks and median total number of doses was 81 vs 50, respectively. In the self‐administration and HCP groups, respectively: 95.0% and 100.0% of patients achieved ≥1 platelet response (defined as weekly platelet count ≥50 × 10^9^/L without rescue medication in previous 4 weeks); the median percentage of weeks with a response was 94.5% and 95.9%; and rescue medication was used in 36.7% and 39.8% of patients. Self‐administration did not adversely affect safety; duration‐adjusted rates for all treatment‐emergent adverse events (TEAEs) and bleeding TEAEs were numerically lower with self‐administration. Romiplostim self‐administration appears effective and well tolerated in eligible patients with ITP.

## 1 INTRODUCTION

Immune thrombocytopenia (ITP) is an autoimmune disorder characterized by low platelet counts (<100 × 10^9^/L) in the absence of other underlying causes of thrombocytopenia, and increased risk of bleeding.^1^, ^2^ In addition to bleeding causing anxiety, patientsʼ work and productivity can also suffer, and many have fatigue, all of which directly impacts their quality of life.^2^, ^3^, ^4^

Romiplostim increases platelet counts through activation of the thrombopoietin receptor, leading to an increase in platelet production.^5^ Romiplostim (Nplate; Amgen, Thousand Oaks, CA, USA) is approved for use in the EU, USA, and other countries for the treatment of adults with ITP (chronic ITP in some non‐US countries, including the EU) who are refractory to first‐line treatments.^6^, ^7^, ^8^ Romiplostim is also indicated for pediatric patients aged ≥1 year with chronic ITP (EU), or those with ITP for ≥6 months (USA) who are refractory to other treatments.

In the EU and other Organization for Economic Cooperation and Development countries (but not North America), romiplostim has been approved for self‐administration by patients and caregivers since the time of its initial registration.^7^ Provided patients are able to have their platelet counts monitored every 4 weeks by a healthcare professional (HCP), romiplostim self‐administration is convenient and eliminates the need for weekly visits to the clinic.^9^ EU recommendations state that patients may self‐administer romiplostim if they maintain a stable platelet count ≥50 × 10^9^/L for at least 4 weeks, and receive adequate training.^7^, ^10^ Once patients begin self‐administration, monitoring of platelet count and a full blood count is carried out on a 4‐weekly basis.^10^

An early study investigating romiplostim self‐administration demonstrated comparable efficacy and safety with physician administration of romiplostim.^11^, ^12^ This was later confirmed in an integrated analysis of three phase 3 open‐label studies,^11^, ^12^, ^13^, ^14^ which supported the EU approval of romiplostim self‐administration.^9^ Further studies have investigated patient outcomes before and after the initiation of romiplostim self‐administration, and found that platelet responses were safely maintained in the majority of patients.^15^, ^16^

The present study builds upon existing evidence to provide a more comprehensive analysis of romiplostim self‐administration. We describe an integrated efficacy and safety analysis of five clinical studies in which romiplostim self‐administration was used in adult patients. Furthermore, we compare patients who self‐administered romiplostim with those patients who had all their doses administered by an HCP and who had received a stable dose for ≥5 consecutive weeks, and who had achieved a weekly platelet count ≥50 × 10^9^/L (to provide a meaningful comparator).

The results of this study should provide a clearer understanding of the safety and efficacy of romiplostim self‐administration in eligible, adequately trained patients with ITP.

## 2 METHODS

### 2.1 Study design

Data were integrated from five romiplostim trials in patients with ITP aged ≥18 years in which self‐administration of romiplostim was permitted (Table 1).^12^, ^13^, ^14^, ^17^, ^18^ All patients received weekly subcutaneous injections of romiplostim with dose adjustments based on platelet count. Eligibility for self‐administration in all trials, at the investigatorsʼ discretion, required platelet counts ≥50 × 10^9^/L to be achieved without romiplostim dose adjustment for ≥3 or ≥4 consecutive weeks (Table 1). Patients could continue with self‐administration provided their romiplostim dose remained stable, as assessed by platelet counts every 4 weeks. Where dose adjustment was needed, patients were required to return to weekly clinic visits until the dose stabilized. All studies were conducted with institutional review board or independent ethics committee approval and all patients provided written informed consent.

**Table 1:** Studies included in the integrated analysis

Study number (ClinicalTrials.gov identifier)	Phase	Design	Duration of treatment	Intervention	Total number of patients	Number of self‐administration patients	Criteria for self‐administration^a^
**Controlled study**							
20 060 131 (NCT00415532)^13^	3b	Randomized, open‐label	52 weeks	Romiplostim	154	109^b^	Stable dose of romiplostim (to achieve target platelet count 50‐200 × 10^9^/L) for ≥3 consecutive weeks
				SOC	75	–	–
**Uncontrolled studies**							
20 030 213 (NCT00116688)^12^	3	Open‐label, extension	≤5 years	Romiplostim	292	239	Stable dose of romiplostim (to achieve target platelet count 50–200 × 10^9^/L) for ≥3 consecutive weeks
20 080 009 (NCT00907478)^17^	4	Open‐label, Single‐arm	≤3 years	Romiplostim	169	112	Stable dose of romiplostim (to achieve target platelet count 50–200 × 10^9^/L) for ≥4 consecutive weeks
20 080 435 (NCT01143038)^18^	2	Open‐label, single‐arm	≤12 months	Romiplostim	75	43	Stable dose of romiplostim (to achieve target platelet count ≥50 × 10^9^/L) for ≥4 consecutive weeks
20 040 209 (NCT00508820)^14^	3b	Open‐label, single‐arm	≤4 years	Romiplostim	407	215	Stable dose of romiplostim (to achieve target platelet count ≥50 × 10^9^/L) for ≥4 consecutive weeks

Abbreviation: SOC, standard of care.

a At the investigatorʼs discretion.

b Romiplostim‐treated patients from Study 20 060 131 could be rolled over into Study 20 030 213 (extension study).

### 2.2 Study sample

The analysis set comprised the self‐administration group and the HCP comparator group. The self‐administration group included any patients who self‐injected romiplostim in any of the studies included in the integrated analysis. For these patients, the index date (day 1) was the first day a subject self‐administered romiplostim across all studies. The HCP group included patients who had received a stable dose of romiplostim for ≥5 consecutive weeks and who had achieved a platelet count ≥50 × 10^9^/L. Patients in this group never self‐injected and continued to attend the clinic for administration of romiplostim. The index date for these patients was the first instance of a fifth consecutive stable dose across all studies. For the HCP group, the requirement for ≥5 consecutive stable doses was specifically chosen to provide an appropriate index date to enable comparisons with the self‐administration group. Although patients in the HCP group did not self‐administer, for those who had stable doses for 4 weeks (stipulated as the minimum for self‐administration in three of the five studies) the fifth dose would represent the equivalent time point at which self‐administration could have started.

### 2.3 Objectives

The primary objectives of the integrated analysis were to examine the efficacy and safety of romiplostim self‐administration.

The efficacy analysis evaluated platelet response to romiplostim, defined as a weekly platelet count ≥50 × 10^9^/L without the use of any rescue medication within the 4 weeks preceding platelet measurement. This was assessed at 4‐week intervals and excluded platelet counts measured following on‐study splenectomy. Rescue medication was defined as any medication or transfusion used for the purposes of increasing platelet counts. Analysis of platelet response included evaluation of the incidence of platelet response, percentage of time with a platelet response, and percentage of patients with platelet counts <50 and ≥50 × 10^9^/L. Also included in the efficacy analysis was the incidence of rescue medication use. In addition, the total number of romiplostim doses was assessed. Finally, the number of dose adjustments (defined as observing different stable doses following ≥1 dose change in between) within 8 weeks and 6 months of the index date, as well as the duration‐adjusted rate of dose adjustment (number of dose adjustments per 100 subject‐weeks) were evaluated. Dosing algorithms used for each study are described in Table S1.

Safety data were collected throughout the entirety of each study and comprised the nature, frequency, severity, seriousness and relationship to treatment of treatment‐emergent adverse events (TEAEs). The TEAEs that led to withdrawal from romiplostim treatment or study withdrawal were recorded. Events of special interest included hypersensitivity, cardiac disorders, drug‐related hepatic disorders, thrombotic/thromboembolic events, hemorrhages, immunogenicity, malignancies, myelofibrosis, pulmonary disorders, renal disorders, thrombocytosis, cytopenias, anemia, and leukocytosis. Also, TEAEs were coded according to Medical Dictionary for Regulatory Activities (MedDRA) version 20.1 by Standardized MedDRA Query (SMQ) and their preferred term. Bleeding events were identified using a pre‐defined list of preferred terms from the “Hemorrhages” (narrow search) SMQ.

### 2.4 Data management and analytical methods

Data were analyzed using descriptive statistics with no hypothesis testing. Continuous variables were described by number (n), mean, SD, median, interquartile range (IQR; Q1‐Q3), minimum and maximum. Numbers and percentages were used for categorical variables.

Safety parameters were adjusted for differences in romiplostim treatment duration in the self‐administration and HCP groups and presented as duration‐adjusted event rates (calculated as: [total number of events/total duration in patient‐years] × 100).

## 3 RESULTS

### 3.1 Patients

The analysis set comprised 754 patients, of which 621 patients were in the self‐administration group and 133 were in the HCP group. Baseline demographics and patient characteristics are shown in Table 2. While statistical testing was not performed, there was a numerical trend for patients in the self‐administration group to be younger than those in the HCP group (median age of 53 vs 58 years, respectively) and with a longer median time since primary ITP diagnosis (3.7 vs 2.5 years, respectively). Median baseline platelet counts at ITP diagnosis between the groups were comparable (19.0 × 10^9^/L in the self‐administration group vs 20.0 × 10^9^/L in the HCP group).

**Table 2:** Demographics and baseline characteristics

Characteristic	Patient group	
	Self‐administration (n = 621)	HCP (n = 133)
Female, n (%)	383 (61.7)	83 (62.4)
Age, median (range), years	53 (18‐93)	58 (18‐89)
White/Caucasian, n (%)	566 (91.1)	119 (89.5)
Weight, median (IQR), kg	77.8 (66.0‐91.8)	74.0 (63.0‐88.0)
Baseline platelet count at ITP diagnosis, median (IQR), ×10^9^/L	19.0 (11.0‐31.0)	20.0 (12.0‐33.0)
Time since primary ITP diagnosis, median (IQR), years	3.7 (0.9‐9.7)	2.5 (0.5‐7.3)
Splenectomy performed, n (%)	211 (34.0)	37 (27.8)
Concurrent ITP therapy, n (%)	164 (26.4)	35 (26.3)

Abbreviations: HCP, healthcare professional; IQR, interquartile range; ITP, primary immune thrombocytopenia.

A higher proportion of patients in the self‐administration group (38%) vs the HCP group (21%) came from a long‐term, open‐label extension study, which analyzed the clinical effects of up to 5 years of romiplostim dosing.^12^ Median exposure to romiplostim was 89 weeks (IQR 52‐155) in the self‐administration group compared with 52 weeks (IQR 27‐116) in the HCP group. Patients within the self‐administration group received a median of 81 doses (IQR 51‐142) vs 50 doses (IQR 24‐87) in the HCP group. The median weekly romiplostim dose for patients in the self‐administration group was 3.6 μg/kg, compared with a slightly lower dose of 3.1 μg/kg in the HCP group.

One patient (0.2%) in the self‐administration group and 27 patients (20.3%) in the HCP group experienced dose adjustment within 8 weeks of the index date. Within 6 months of the index date, 149 (24.0%) patients in the self‐administration group and 63 (47.4%) patients in the HCP group underwent dose adjustment. From 4 weeks to 1 week prior to the index date (weeks −4 to −1), duration‐adjusted dose adjustment rates were numerically higher in the self‐administration group compared with the HCP group (1.13 and 0.72 per 100 patient‐weeks, respectively). In the weeks following the index date (week 1 until the end of treatment), dose adjustment rates were 1.24 vs 1.60 per 100 patient‐weeks in the self‐administration vs HCP groups, respectively.

Overall, the median number of visits for assessment of platelet counts was 47 vs 53 in the self‐administration group and HCP group, respectively.

### 3.2 Efficacy

During weeks −4 to −1, 86.9% (95% exact binomial confidence interval [CI]: 84.0%‐89.5%) of the self‐administration group and 100.0% of the HCP group (95% CI: 97.3%‐100.0%) achieved a platelet response (weekly platelet count ≥50 × 10^9^/L without any use of rescue medication) at least once. From week 1 until end of treatment, 95.0% (95% CI: 93.0%‐96.6%) of the self‐administration group and 100.0% (95% CI: 97.3‐100.0%) of patients in the HCP group achieved platelet response (weekly platelet count ≥50 × 10^9^/L without any use of rescue medication in the prior 4 weeks) at least once.

**Figure 1:**
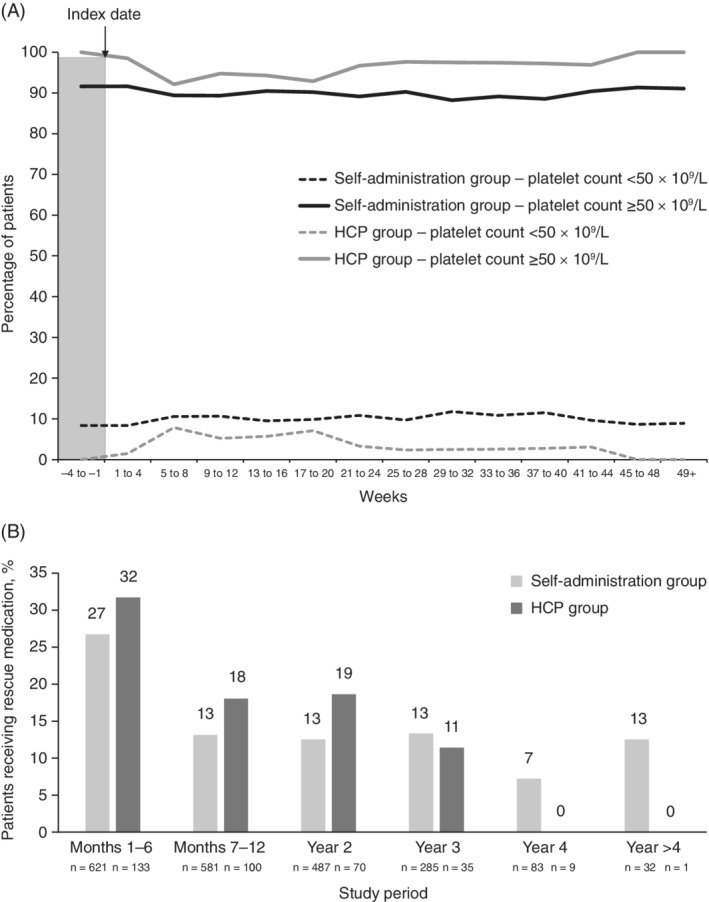
A, Proportions of patients in platelet count categories immediately prior to (Weeks −4 to −1) and during the study period; *, B, Rescue medication requirements by study period. *Patients in the self‐administration group were required to have ≥3 consecutive stable doses (maintaining platelet counts at ≥50 × 10^9^/L) ‐ therefore, during Weeks −4 to –1 some patients in the self‐administration group may have had counts <50 × 10^9^/L on one of their visits. HCP, healthcare professional dosing; SA, self‐administration

The proportion of patients with platelet counts ≥50 × 10^9^/L from week 1 to end of treatment ranged between a minimum of 88.2% and a maximum of 91.6% in the self‐administration group, and 92.1% and 100.0% in the HCP group (Figure 1A). The majority of patients in both groups had platelet levels between 50 and 200 × 10^9^/L during treatment. No more than 3.5% of patients in the self‐administration group and 3.1% of patients in the HCP group had platelet counts of <20 × 10^9^/L at any visit. Similarly, no more than 2.2% of self‐administration patients and 4.7% of HCP‐dosed patients had platelet levels of >400 × 10^9^/L. The percentage of weeks in which patients had a platelet response from week 1 to end of treatment was numerically comparable in the two groups; for the self‐administration group, patients experienced a median of 94.5% (IQR: 73.5%‐100.0%) of these weeks with a platelet response (≥50 × 10^9^/L) compared with 95.9% (IQR: 81.3%‐100.0%) of weeks for patients in the HCP group. Rescue medication was most commonly given during the first 6 months of the study period (Figure 1B). Overall, a similar proportion of patients in each group received rescue medication at some point (36.7% [n = 228] in the self‐administration group vs 39.8% [n = 53] in the HCP group). Corticosteroids, administered with or without intravenous immunoglobulins, were given most frequently. Corticosteroids were administered to 79.8% (182/228) and 71.6% (38/53) of patients who required rescue medication in the self‐administration and HCP groups, respectively. Similar proportions of patients in the self‐administration group and HCP group received immunoglobulin (39.0% [89/228] and 37.7% [20/53], respectively), or platelet transfusion (11.0% [35/228] and 11.3% [6/53], respectively) rescue medication.

### 3.3 Safety

Any TEAEs were reported in 94.4% (n = 586) and 96.2% (n = 128) of self‐administration and HCP‐dosed groups, respectively. When adjusted for romiplostim duration, the rate of observed TEAEs was numerically lower in the self‐administration group (735.7 events/100 patient‐years compared with 918.4 events/100 patient‐years in the HCP group) (Table 3). The overall incidence of grade ≥ 3 adverse events (AEs) and serious adverse events (SAEs) were similar for both the self‐administration and HCP groups (42.7% vs 41.4% of patients experienced grade ≥ 3 AEs and 34.6% vs 34.6% SAEs, respectively). Duration‐adjusted rates for both grade ≥ 3 AEs and SAEs were numerically lower in the self‐administration vs HCP group (Table 3).

**Table 3:** Overview of treatment‐emergent adverse events (TEAEs)

	Incidence number of patients (%)^a^		Duration‐adjusted rates number of events (events/100 patient‐years)^b^	
	Self‐administration (n = 621)	HCP (n = 133)	Self‐administration patient‐years = 1019.9 (n = 621)	HCP patient‐years = 138.2 (n = 133)
**All TEAEs**	586 (94.4)	128 (96.2)	7503 (735.7)	1269 (918.4)
Grade ≥3	265 (42.7)	55 (41.4)	595 (58.3)	128 (92.6)
SAEs	215 (34.6)	46 (34.6)	360 (35.3)	86 (62.2)
Leading to withdrawal of romiplostim	37 (6.0)	15 (11.3)	39 (3.8)	8 (5.8)
Leading to study discontinuation	30 (4.8)	16 (12.0)	25 (2.5)	8 (5.8)
Fatal TEAEs	15 (2.4)	10 (7.5)	13 (1.3)	5 (3.6)
**Treatment‐emergent bleeding events**	278 (44.8)	53 (39.8)	1313 (128.7)	185 (133.9)
Grade ≥3	41 (6.6)	13 (9.8)	96 (9.4)	21 (15.2)
SAEs	30 (4.8)	8 (6.0)	47 (4.6)	12 (8.7)
Leading to withdrawal of romiplostim	2 (0.3)	0	4 (0.4)	0
Leading to study discontinuation	0	0	0	0
Fatal bleeding TEAEs	1 (0.2)	0	1 (0.1)	0

Abbreviations: AE, adverse event; HCP, healthcare‐professional‐dosed group; SAE, serious adverse event; TEAE, treatment‐emergent adverse event.

a AEs starting after the first dose of investigational product.

b Index Week 1 to end of treatment.

The most frequent TEAEs were headache (38.5%), nasopharyngitis (30.9%), and arthralgia (26.6%) in the self‐administration group and headache (30.1%), fatigue (22.6%), and nausea (22.6%) in the HCP group (Table S2). The most common SAEs in both groups were thrombocytopenia (5.8% self‐administration vs 4.5% HCP) and pneumonia (2.7% vs 1.5%) (Table S2). Duration‐adjusted event rates were similar between self‐administration and HCP patients for the most common TEAEs and SAEs (Table S2).

While duration‐adjusted rates of bleeding TEAEs were similar between groups (128.7 vs 133.9 events/100 patient‐years in the self‐administration vs HCP groups, respectively), the duration‐adjusted grade ≥3 bleeding TEAEs and SAEs associated with bleeding were numerically lower for self‐administration, vs HCP‐dosed patients at 9.4 vs 15.2 (grade ≥ 3), and 4.6 vs 8.7 (SAEs) events/100 patient‐years, respectively (Table 3).

Thrombotic/thromboembolic events were observed in 7.1% of self‐administration patients and 9.8% of the HCP group; duration‐adjusted values were 4.8 and 8.8 events/100 patient‐years, respectively (Table S3). The only thrombotic event occurring in ≥1% of patients in both treatment groups was deep vein thrombosis (9/621 [1.4%] and 2/133 [1.5%] patients in the self‐administration and HCP groups, respectively). Most of the patients who had thrombotic/thromboembolic events had at least one weekly platelet response (43 out of 44 of the self‐administration patients and all of the 13 HCP patients).

The TEAEs leading to withdrawal of romiplostim or discontinuation of study occurred numerically less frequently in the self‐administration group (6.0% and 4.8%, respectively) compared with the HCP group (11.3% and 12.0% respectively; Table 3). Fatal TEAEs were reported in 15 patients (2.4%) in the self‐administration group and 10 patients (7.5%) in the HCP group (Table 3), of which three were reported as treatment‐related by the investigator. Two were in the self‐administration group (unstable angina, myocardial infarction) and one in the HCP‐dosed group (intestinal ischemia) (Table S4). No errors in medication were reported for either group.

## 4 DISCUSSION

This *post‐hoc* analysis, integrating data from five clinical trials of romiplostim, suggests that romiplostim self‐administration has comparable efficacy and safety to that of romiplostim administered by an HCP.

Most patients responded well to romiplostim treatment and attained their target platelet counts in the 4 weeks preceding self‐administration, with response rates generally exceeding 90%. These high platelet response rates were maintained after the commencement of self‐administration and were similar to those in the HCP group. These response rates are comparable to those observed in a study of patients undergoing long‐term treatment with romiplostim for ITP, which reported platelet responses in 87% of patients (N = 142), two‐thirds of whom self‐administered their medication.^11^ Additionally, the use of rescue medication was similar in both groups (36.7% self‐administration group vs 39.8% HCP group) and is aligned with other studies of romiplostim.^11^

Patients who self‐administer romiplostim do not seem to be at additional risk of thrombocytosis or thrombocytopenia. Although not accurate predictors of thrombosis or bleeding, thrombocytosis and thrombocytopenia are sensitive indicators of dosing accuracy. In our analysis, very few patients (less than 4% in each group) had low platelet counts of <20 × 10^9^/L at any visit. Similarly, only 2.2% of self‐administration patients and 4.7% of HCP‐dosed patients had high platelet levels (>400 × 10^9^/L).

Dose adjustments appeared to be numerically less common with self‐administration than HCP administration between week 1 and end of treatment. The proportion of patients experiencing dose adjustments within 6 months of the index date in the self‐administration group (24%) was lower than that observed in an open‐label extension study of romiplostim self‐administration where 35% of patients (N = 292) had one or more dose adjustments over the first 6 months of self‐administration.^16^ Overall, these data suggest that dose adjustments are not negatively impacted by self‐administration of romiplostim. The numerically higher rate of dose adjustments in the HCP arm may also be reflective of the possibility that patients who tend to require dose adjustments would be less likely to be allowed to self‐administer, thus receiving HCP administration only, making them eligible to be included in the HCP population. Additionally, patients in the HCP group attended numerically more visits for platelet assessments than self‐administration patients (53 vs 47, respectively), giving more opportunity for dose adjustments in the former group.

Self‐administration did not appear to affect the safety profile of romiplostim compared with patients in the HCP group. The most commonly observed TEAEs in the self‐administration group ‐ headache, nasopharyngitis, and arthralgia ‐ are congruent with those reported in open‐label extension studies of extended romiplostim treatment.^11^, ^16^ The rates of SAEs (34.6% in both self‐administration and HCP‐dosed patients) are also comparable to those seen in the long‐term dosing study (31% of patients reported a SAE).^11^ Overall, 7.1% of patients in the self‐administration group experienced a thrombotic/thromboembolic event compared with 9.8% in the HCP group. Duration‐adjusted rates of TEAEs were numerically lower in self‐administration compared with HCP‐dosed patients (735.7 vs 918.4 events/100 patient‐years). Additionally, while statistical testing was not performed, there was a trend for treatment‐related TEAEs leading to drug withdrawal, study discontinuation and fatal events to be numerically less frequent in the self‐administration group compared with the HCP group when adjusted for treatment duration. These apparent reductions in TEAE incidences in the self‐administration group may be due to several reasons. Firstly, due to the non‐randomized allocation of patients to the self‐administration and HCP dosing groups, clinicians may have offered self‐administration to “fitter” patients; indeed, median age was lower in the self‐administration group. This may also explain the lower percentage of patients requiring dose adjustments in the self‐administration group. Additionally, an important factor to consider is the reduced number of clinic visits attended by the self‐administration group, at which monitoring could be carried out and where TEAEs would be reported. For example, patients in the self‐administration group attended numerically fewer visits for platelet counts than the HCP group. Accuracy could have been affected by patientsʼ recall of events that may have occurred several weeks before. Although this may be a contributing factor to the lower rates of TEAEs and dose adjustments observed in the self‐administration group, efficacy of self‐administration and overall safety were not largely affected and still support the use of romiplostim self‐administration. Overall, the TEAE profile of romiplostim in self‐administration patients from this study was similar to that from the earlier integrated analysis of self‐administration in three clinical trials,^9^ and no new safety concerns were identified when compared with published product information for romiplostim.^12^, ^13^

Patients with ITP are known to visit their physician more often and to have poorer work and productivity scores than healthy age‐ and sex‐matched controls.^3^ Our results suggest that self‐administration of romiplostim may be a practical option for the treatment of ITP. Expanding the availability of self‐administration in romiplostim‐treated patients may reduce the need for clinic visits by patients (as demonstrated in our analysis by the numerically lower number of visits for platelet count assessments in the self‐administration group vs the HCP group) and could minimize absenteeism from work or education.^16^

The feasibility of expanding the use of romiplostim self‐administration is strengthened by the availability of dedicated Home Administration Training (HAT) materials for HCPs and patients that are distributed with self‐administration kits.^10^, ^19^ These include a guide to self‐administration, step‐by‐step instructions (including preparing the vial for use, correct reconstitution of drug ensuring complete dissolution, accurate measurement of the injection volume, selection and preparation of the injection site, and subcutaneous injection), a DVD on self‐administration, preparation mat, and self‐administration diary, with the aim of ensuring that patients are administering the correct dose in a safe manner and remain aware of all doses given. Patients are also encouraged to report any side effects directly online with their appropriate local pharmacovigilance agency and the manufacturer. The effect of including HAT materials with self‐administration kits has been evaluated as part of a multicenter observational study.^19^ Among 40 patients/caregivers enrolled across 12 study centers, 35 (87.5%) administered romiplostim correctly following use of HAT materials and no injection errors were recorded.^19^ This supports the use of HAT resources as a risk minimization tool.^19^ In clinical studies,^11^, ^12^, ^14^, ^15^, ^16^ between 3%‐24% of patients initiating self‐administration of romiplostim changed to HCP‐administration. In the majority of these cases, this was due to an administrative/investigator decision. Other reasons included patient request and in a small minority of cases (~1% of patients who started self‐administration) due to non‐adherence.^11^, ^12^, ^14^, ^15^, ^16^ Data reporting compliance with romiplostim self‐administration in real‐world clinical settings are limited. In an observational study of romiplostim in real‐world European clinical practice, 125 of 340 (36%) patients self‐administered at least one dose. The safety and efficacy in these patients were similar to those who had received HCP‐administered romiplostim.^20^

Our analysis provides some insights on which patients tend to be selected by HCPs for self‐administration of romiplostim. While statistical testing was not performed in this descriptive analysis, the self‐administration group had a trend towards a lower median age than the HCP group (53 vs 58 years, respectively), suggesting that younger patients may be deemed to be more suitable. Additionally, patients in the self‐administration group appeared to be more experienced with managing their condition than those in the HCP group, with a numerically longer median time since primary diagnosis of ITP (3.7 vs 2.5 years in the HCP group), and a higher incidence of splenectomy (34.0% vs 27.8% in the HCP group).

By integrating data from five romiplostim self‐administration trials, this study provides a comprehensive analysis of self‐administration compared with HCP administration in adult patients with a stable platelet count for ≥3 consecutive weeks. This study has an important strength compared with previous studies assessing romiplostim self‐administration,^9^, ^15^, ^16^ because patients in both the self‐administration and HCP groups were selected for dose stability at the index date. Limitations of the study include the retrospective, non‐randomized study design, analysis of trials based on different designs, the descriptive nature of the analyses, differing numbers of observation points, recall bias, potentially limited generalizability of the study population to a real‐world population due to exclusion criteria of the trials, and the absence of assessments of the impact of self‐administration on quality of life (not all of the included studies evaluated quality of life).

In conclusion, these findings suggest that romiplostim self‐administration is efficacious and does not compromise safety in patients with ITP who have achieved stable platelet counts ≥50 × 10^9^/L under HCP supervision and who are willing to undergo training. Romiplostim self‐administration may lead to time savings for patients and HCPs and, therefore, potential cost savings for healthcare systems.

## CONFLICT OF INTEREST

D.J.K. has received research funding from Actelion (Syntimmune), Agios, Alnylam, Amgen, Argenx, BristolMyers Squibb, Kezar, Principia, Protalex, Rigel, Takeda (Bioverativ), and UCB, and acted as a consultant for Actelion (Syntimmune), Agios, Alnylam, Amgen, Argenx, BristolMyers Squibb, Caremark, Daiichi Sankyo, Dova, Kyowa‐Kirin, Merck, Sharp & Dohme, Momenta, Novartis, Pfizer, Platelet Disorder Support Association, Principia, Protalex, Protalix, Rigel, Rubius, Sanofi, Genzyme, Shionogi, Shire, Takeda (Bioverativ), UCB, Up‐To‐Date, and Zafgen. D.M.A. has received research funding from Novartis and BristolMyers Squibb, and acted as a consultant for Rigel, Principia, Novartis, and Amgen. F.R. has acted as a consultant for, received lecture fees from, and received research funding from Amgen and Novartis. A.J. has participated on advisory boards, and received travel grants and speakerʼs fees for AbbVie, Amgen, Celgene, Janssen, Novartis, Roche, and Sanofi Genzyme. D.S. has acted as a consultant for and participated in speakerʼs bureau for Amgen. R.B. has been part of a speaker faculty (honoraria declined) for Amgen and Novartis, and acted as a principal investigator in clinical trials for Amgen, Novartis, CSL, BristolMyers Squibb, Principia Pharma, and Rigel. A.N. has acted as a consultant for Amgen, Angle, Argenx, Dova, Novartis, Ono, Rigel, and Shionogi, received research funding from Amgen, Novartis, and Rigel, received honoraria from Amgen, Angle, Argenx, Dova, Novartis, Ono, Rigel, and Shionogi, and provided paid expert testimony for Argenx and Rigel. J.M. has received research funding from Amgen, Dova Pharmaceuticals and Rigel. K.M. and R.O. are employed by Amgen.

## DATA SHARING STATEMENT

There is a plan to share data. This may include de‐identified individual patient data for variables necessary to address the specific research question in an approved data‐sharing request; also related data dictionaries, study protocol, statistical analysis plan, informed consent form, and/or clinical study report. Data sharing requests relating to data in this manuscript will be considered after the publication date and (a) this product and indication (or other new use) have been granted marketing authorization in both the US and Europe, or (b) clinical development discontinues and the data will not be submitted to regulatory authorities. There is no end date for eligibility to submit a data sharing request for these data. Qualified researchers may submit a request containing the research objectives, the Amgen product(s) and Amgen study/studies in scope, endpoints/outcomes of interest, statistical analysis plan, data requirements, publication plan, and qualifications of the researcher(s). In general, Amgen does not grant external requests for individual patient data for the purpose of re‐evaluating safety and efficacy issues already addressed in the product labeling. A committee of internal advisors reviews requests. If not approved, requests may be further arbitrated by a Data Sharing Independent Review Panel. Requests that pose a potential conflict of interest or an actual or potential competitive risk may be declined at Amgenʼs sole discretion and without further arbitration. Upon approval, information necessary to address the research question will be provided under the terms of a data sharing agreement. This may include anonymized individual patient data and/or available supporting documents, containing fragments of analysis code where provided in analysis specifications. Further details are available at the following: https://wwwext.amgen.com/science/clinical-trials/clinical-data-transparency-practices/.

## Supporting information


**Appendix S1**. Supporting information.
