# Supplementary material for: Safety and efficacy of self‐administered romiplostim in patients with immune thrombocytopenia: Results of an integrated database of five clinical trials
Source: Am J Hematol. 2020 Mar 21;95(6):643–51. doi: 10.1002/ajh.25776 (PMC7318268; doi:10.1002/ajh.25776)
Supplement: Supplementary file 1 — Appendix S1. Supporting information. [file AJH-95-643-s001.docx]

# Supplementary Online Material

# Safety and Efficacy of Self-Administered Romiplostim in Patients with Immune Thrombocytopenia: Results of an Integrated Database of Five Clinical Trials

David J. Kuter MD, DPhil,^1^ Donald M. Arnold MD, MSc,^2^ Francesco Rodeghiero MD,^3^ Ann Janssens MD,^4^ Dominik Selleslag MD,^5^ Robert Bird MBBS, FRACP, FRCPA, FRCP,^6^ Adrian Newland MA, FRCP, FRCPath,^7^ Jiri Mayer MD,^8^ Kejia Wang PhD,^9^ Robert Olie DPhil^10^

^1^Hematology Division, Massachusetts General Hospital, Boston, MA, USA; ^2^McMaster Centre for Transfusion Research, Department of Medicine, Michael G DeGroote School of Medicine, McMaster University, Hamilton, ON; and Canadian Blood Services, Hamilton, ON, Canada; ^3^Haematology Project Foundation, affiliated to the Department Haematology, S. Bortolo Hospital, Vicenza, Italy; ^4^Department of Hematology, University Hospitals Leuven, Campus Gasthuisberg, Leuven, Belgium; ^5^Department of Hematology, AZ Sint Jan Brugge, Brugge, Belgium; ^6^Division of Cancer Services, Princess Alexandra Hospital, Brisbane, Australia; ^7^The Pathology Clinical Academic Group, The Royal London Hospital, London, UK; ^8^Department of Internal Medicine, Haematology and Oncology, Masaryk University and University Hospital Brno, Brno, Czech Republic; ^9^Amgen Inc, Thousand Oaks, CA, USA; ^10^Amgen (Europe) GmbH, Rotkreuz, Switzerland

# Supplementary Online Material

| **Supplementary Table S1.** Romiplostim dosing algorithms used in each study | |
| --- | --- |
| **Study number (ClinicalTrials.gov identifier)** | **Action** |
| **20060131 (NCT00415532)** | |
| Platelet count (x10^9^/L)  <30 | Dose will be increased by 1 μg/kg every week |
| 30 to 450 | Dose may be adjusted (increased or decreased) in increments of 1 μg/kg at the investigator’s discretion no more frequently than every 2 weeks |
| >450 | Dose will be held. The dose will be reduced by 1 μg/kg at the next scheduled dosing day when platelet count has fallen to <200 ×10^9^/L |
| **20030213 (NCT00116688)** | |
| Platelet count (x10^9^/L)  <50 | Dose should be increased by 1 μg/kg weekly. Dose may be increased every week |
| 50 to 400 | Dose may be adjusted by 1 μg/kg (increased or reduced) at the physician’s discretion targeting a platelet count of 50 to 200 × 10^9^/L (50–250 × 10^9^/L before October 2007). Dose may be adjusted every 2 weeks |
| >400 | Dose should be withheld. The dose may be reduced by 1 μg/kg on the next scheduled dosing day that the platelet count is <200 × 10^9^/L |
| **20080009 (NCT00907478)** | |
| Platelet count (x10^9^/L)  50 to 200 | Weekly adjustment in increments of 1 μg/kg up to a maximum of 10 μg/ kg to target platelet counts between 50 and 200 × 10^9^/L |
| **20080435 (NCT01143038)** | |
| Platelet count (x10^9^/L)  <50 | Increase dose by 1 µg/kg each week |
| 50 to 200 | Dose remains constant |
| >200 to <400 | After the platelet count remains in this range for 2 consecutive weeks, reduce the dose by 1 µg/kg |
| ≥400 | Withhold the dose, and reduce the dose by 1 µg/kg on the next scheduled day of dosing when platelet count falls below 200 × 10^9^/L |
| **20040209 (NCT00508820)** | |
| Platelet count (x10^9^/L)  ≤20 | Patient considered non-responder and discontinued from the study if they have received 10 μg/kg for 4 consecutive weeks unless the investigator felt the patient was benefitting clinically and the sponsor gave permission for the patient to continue on the study |
| <50 | Dose increased by 1 μg/kg/week when platelet counts below 50 × 10^9^/L. Dose could be increased every week, to a maximum of 10 μg/kg |
| >200 | Dose reduced by 1 μg/kg after 2 consecutive weeks of platelet counts above 200 × 10^9^/L |
| >400 | Next scheduled dose withheld; thereafter, dose reduced by 1 μg/kg on the next scheduled dosing day platelet count was below 200 × 10^9^/L |

| **Supplementary Table S2.** Most frequently reported treatment-emergent adverse events (TEAEs), serious adverse events (SAEs) and bleeding events in terms of incidence and rates adjusted for duration of treatment. | | | | |
| --- | --- | --- | --- | --- |
|  | **Incidence Number of patients (%)*** | | **Duration-adjusted rates Number of events (events/100 patient-years)^†^** | |
|  | **Self-administration (n=621)** | **HCP**  **(n=133)** | **Self-administration patient-years=1289.4**  **(n=621)** | **HCP patient-years=203.5**  **(n=133)** |
| **Most frequent TEAEs**^‡^ | | | | |
| Headache | 239 (38.5) | 40 (30.1) | 634 (49.2) | 84 (41.3) |
| Nasopharyngitis | 192 (30.9) | 25 (18.8) | 347 (26.9) | 56 (27.5) |
| Arthralgia | 165 (26.6) | 25 (18.8) | 293 (22.7) | 38 (18.7) |
| Fatigue | 155 (25.0) | 30 (22.6) | 326 (25.3) | 42 (20.6) |
| Epistaxis | 147 (23.7) | 24 (18.0) | 366 (28.4) | 42 (20.6) |
| Contusion | 142 (22.9) | 27 (20.3) | 456 (35.4) | 66 (32.4) |
| Upper respiratory tract infection | 133 (21.4) | 17 (12.8) | 248 (19.2) | 24 (11.8) |
| Diarrhea | 127 (20.5) | 26 (19.5) | 216 (16.8) | 46 (22.6) |
| Nausea | 127 (20.5) | 30 (22.6) | 210 (16.3) | 47 (23.1) |
| Cough | 116 (18.7) | 29 (21.8) | 185 (14.3) | 47 (23.1) |
| Pain in extremity | 109 (17.6) | 28 (21.1) | 169 (13.1) | 51 (25.1) |
| **Most frequent SAEs**^§^ | | | | |
| Thrombocytopenia | 36 (5.8) | 6 (4.5) | 51 (4.0) | 7 (3.4) |
| Pneumonia | 17 (2.7) | 2 (1.5) | 21 (1.6) | 4 (2.0) |
| Hemorrhage | 8 (1.3) | 0 | 8 (0.6) | 0 |
| Gastrointestinal hemorrhage | 7 (1.1) | 1 (0.8) | 8 (0.6) | 1 (0.5) |
| Pulmonary embolism | 7 (1.1) | 1 (0.8) | 7 (0.5) | 2 (1.0) |
| Abdominal pain | 6 (1.0) | 1 (0.8) | 7 (0.5) | 1 (0.5) |
| Cardiac failure | 6 (1.0) | 0 | 7 (0.5) | 0 |
| Deep vein thrombosis | 6 (1.0) | 2 (1.5) | 7 (0.5) | 2 (1.0) |
| Epistaxis | 6 (1.0) | 4 (3.0) | 8 (0.6) | 7 (3.4) |
| Myocardial infarction | 6 (1.0) | 2 (1.5) | 6 (0.5) | 2 (1.0) |
| Syncope | 4 (0.6) | 2 (1.5) | 5 (0.4) | 2 (1.0) |
| Atrial fibrillation | 3 (0.5) | 2 (1.5) | 3 (0.2) | 2 (1.0) |
| Cardiac failure congestive | 3 (0.5) | 2 (1.5) | 5 (0.4) | 8 (3.9) |
| Cerebrovascular accident | 3 (0.5) | 2 (1.5) | 3 (0.2) | 2 (1.0) |
| Renal failure | 3 (0.5) | 2 (1.5) | 3 (0.2) | 2 (1.0) |
| **Most frequent bleeding TEAEs**^‖^ | | | | |
| Epistaxis | 147 (23.7) | 24 (18.0) | 366 (28.4) | 42 (20.6) |
| Contusion | 142 (22.9) | 27 (20.3) | 456 (35.4) | 66 (32.4) |
| Petechiae | 112 (18.0) | 19 (14.3) | 197 (15.3) | 38 (18.7) |
| Hematoma | 66 (10.6) | 14 (10.5) | 156 (12.1) | 23 (11.3) |
| Gingival bleeding | 65 (10.5) | 14 (10.5) | 119 (9.2) | 22 (10.8) |
| Ecchymosis | 41 (6.6) | 9 (6.8) | 84 (6.5) | 13 (6.4) |
| Mouth hemorrhage | 37 (6.0) | 9 (6.8) | 69 (5.4) | 11 (5.4) |

*Adverse events starting after the first dose of investigational product.

**^†^**Index Week 1 to end of treatment.

^‡^Experienced by ≥20% of patients in either group.

^§^Experienced by ≥1% of patients in either group. Table excludes immune thrombocytopenia (ITP), which was reported as a SAE in 12 (1.9%) and 3 (2.3%) of patients in the self-administration and HCP groups, respectively. Excluded from the table since all patients in the study had ITP.

^‖^Experienced by ≥5% of patients in either group. Table excludes immune thrombocytopenia (ITP), which was reported as a TEAE in 51 (8.2%) and 10 (7.5%) of patients in the self-administration and HCP groups, respectively. Excluded from the table since all patients in the study had ITP.

HCP, healthcare professional dosing; SAE, serious adverse event; TEAE, treatment-emergent adverse event.

| **Supplementary Table S3.** Most frequently reported, treatment-emergent thrombotic/thromboembolic adverse events and hemorrhage adverse events (categorized by standardized MedDRA query), in terms of incidence and rates adjusted for duration of treatment*. | | | | | |
| --- | --- | --- | --- | --- | --- |
| **SMQ, n (%)** | **Incidence Number of patients (%)** | | | **Duration-adjusted rates Number of events (events/100 patient-years)** | |
|  | **Self-administration (n=621)** | **HCP (n=133)** | **Self-administration patient-years=1289.4 (n=621)** | | **HCP patient-years=203.5 (n=133)** |
| **Thrombotic/Thromboembolic Events**^†^ | 44 (7.1) | 13 (9.8) | 62 (4.8) | | 18 (8.8) |
| Deep vein thrombosis | 9 (1.4) | 2 (1.5) | 13 (1.0) | | 2 (1.0) |
| Pulmonary embolism | 8 (1.3) | 1 (0.8) | 8 (0.6) | | 2 (1.0) |
| Cerebrovascular accident | 3 (0.5) | 2 (1.5) | 3 (0.2) | | 2 (1.0) |
| Portal vein thrombosis | 2 (0.3) | 3 (2.3) | 2 (0.2) | | 3 (1.5) |
| Transient ischemic attack | 2 (0.3) | 2 (1.5) | 2 (0.2) | | 2 (1.0) |
| **Hemorrhages**^‡^ | 361 (58.1) | 77 (57.9) | 2063 (160.0) | | 330 (162.1) |
| Epistaxis | 147 (23.7) | 24 (18.0) | 366 (28.4) | | 42 (20.6) |
| Contusion | 142 (22.9) | 27 (20.3) | 456 (35.4) | | 66 (32.4) |
| Petechiae | 112 (18.0) | 19 (14.3) | 197 (15.3) | | 38 (18.7) |
| Hematoma | 66 (10.6) | 14 (10.5) | 156 (12.1) | | 23 (11.3) |
| Gingival bleeding | 65 (10.5) | 14 (10.5) | 119 (9.2) | | 22 (10.8) |
| Ecchymosis | 41 (6.6) | 9 (6.8) | 84 (6.5) | | 13 (6.4) |
| Mouth hemorrhage | 37 (6.0) | 9 (6.8) | 69 (5.4) | | 11 (5.4) |
| *AEs starting after the first dose of investigational product  ^†^Experienced by ≥1% of patients in either group  ^‡^Experienced by ≥5% of patients in either group, excluding immune thrombocytopenia, which was reported as an adverse event in 51 (8.2%) and 10 (7.5%) of patients in the self-administration and HCP groups, respectively. Excluded from the table since all patients in the study had ITP.  HCP, healthcare professional; MedDRA, Medical Dictionary for Regulatory Activities; NR, not reported; SMQ, standardized MedDRA query. | | | | | |
|  | | | | | |

| **Supplementary Table S4.** Overview of fatal treatment-emergent adverse events by standardized MedDRA query, in terms of incidence and rates adjusted for duration of treatment^*^. | | | | |
| --- | --- | --- | --- | --- |
|  | **Incidence Number of patients (%)*** | | **Duration-adjusted rates Number of events (events/100 patient-years)^†^** | |
|  | **Self-administration (n=621)** | **HCP**  **(n=133)** | **Self-administration Patient-years=1289.4 (n=621)** | **HCP Patient-years=203.5 (n=133)** |
| **All fatal TEAEs** | 15 (2.4) | 10 (7.5) | 15 (1.2) | 10 (4.9) |
| Myocardial infarction | 2 (0.3) | 1 (0.8) | 2 (0.2) | 1 (0.5) |
| Unstable angina | 1 (0.2) | 0 | 1 (<0.1) | 0 |
| Cardiac failure | 1 (0.2) | 0 | 1 (<0.1) | 0 |
| Cardiac failure congestive | 1 (0.2) | 1 (0.8) | 1 (<0.1) | 1 (0.5) |
| Cardiac tamponade | 1 (0.2) | 0 | 1 (<0.1) | 0 |
| Death | 1 (0.2) | 0 | 1 (<0.1) | 0 |
| Hepatocellular carcinoma | 1 (0.2) | 0 | 1 (<0.1) | 0 |
| Intracranial venous sinus thrombosis | 1 (0.2) | 0 | 1 (<0.1) | 0 |
| Ischemic stroke | 1 (0.2) | 0 | 1 (<0.1) | 0 |
| Pneumococcal sepsis | 1 (0.2) | 0 | 1 (<0.1) | 0 |
| Pneumonia | 1 (0.2) | 0 | 1 (<0.1) | 0 |
| Renal failure | 1 (0.2) | 1 (0.8) | 1 (<0.1) | 1 (0.5) |
| Subdural hematoma | 1 (0.2) | 0 | 1 (<0.1) | 0 |
| Thrombosis | 1 (0.2) | 0 | 1 (<0.1) | 0 |
| Fungal sepsis | 0 | 1 (0.8) | 0 | 1 (0.5) |
| Intestinal infarction | 0 | 1 (0.8) | 0 | 1 (0.5) |
| Intestinal ischemia | 0 | 1 (0.8) | 0 | 1 (0.5) |
| Malignant lung neoplasm | 0 | 1 (0.8) | 0 | 1 (0.5) |
| Meningitis listeria | 0 | 1 (0.8) | 0 | 1 (0.5) |
| Pneumonia streptococcal | 0 | 1 (0.8) | 0 | 1 (0.5) |
| Pulmonary hemorrhage | 0 | 1 (0.8) | 0 | 1 (0.5) |
| **Treatment-related fatal TEAEs** | 2 (0.3) | 1 (0.8) | 2 (0.2) | 1 (0.5) |
| Unstable angina | 1 (0.2) | 0 | 1 (<0.1) | 0 |
| Myocardial infarction | 1 (0.2) | 0 | 1 (<0.1) | 0 |
| Intestinal ischemia | 0 | 1 (0.8) | 0 | 1 (0.5) |

*AEs starting after the first dose of investigational product.

**^†^**Index Week 1 to end of treatment.

AE, adverse event; HCP, healthcare-professional-dosing group; SAE, serious adverse event; TEAE, treatment-emergent adverse event.
